# Commensal *Staphylococcus aureus* Provokes Immunity to Protect against Skin Infection of Methicillin-Resistant *Staphylococcus aureus*

**DOI:** 10.3390/ijms19051290

**Published:** 2018-04-25

**Authors:** John-Jackson Yang, Ting-Wei Chang, Yong Jiang, Hsin-Jou Kao, Bin-Hao Chiou, Ming-Shan Kao, Chun-Ming Huang

**Affiliations:** 1 Department of Life Sciences, National Central University, Taoyuan 32001, Taiwan; johnjacksonyang@gmail.com; 2 Department of Biomedical Sciences and Engineering, National Central University, Taoyuan 32001, Taiwan; harry155007@gmail.com (T.-W.C.); lulu21522@yahoo.com.tw (H.-J.K.); aimall0115@gmail.com (B.-H.C.); s36424592@yahoo.com.tw (M.-S.K.); 3 Surface Bioadvances Inc., San Diego, CA 92121, USA; yjiang92122@gmail.com; 4 Department of Dermatology, University of California, San Diego. 3525 John Hopkins Court, Rm276, San Diego, CA 92121, USA

**Keywords:** α-hemolysin, fermentation, microbiome, MRSA, *S. aureus*

## Abstract

Unlike USA300, a strain of community-acquired methicillin-resistant *Staphylococcus aureus* (MRSA), commensal *Staphylococcus aureus* (*S. aureus*) bacteria isolated from human skin demonstrated the ability to mediate the glycerol fermentation to produce short-chain fatty acids (SCFAs). Quantitative proteomic analysis of enzymes involved in glycerol fermentation demonstrated that the expression levels of six enzymes, including glycerol-3-phosphate dehydrogenase (GPDH) and phosphoglycerate mutase (PGM), in commensal *S. aureus* are more than three-fold higher than those in USA300. Western blotting validated the low expression levels of GPDH in USA300, MRSA252 (a strain of hospital-acquired MRSA), and invasive methicillin-susceptible *S. aureus* (MSSA). In the presence of glycerol, commensal *S. aureus* effectively suppressed the growth of USA300 in vitro and in vivo. Active immunization of mice with lysates or recombinant α-hemolysin of commensal *S. aureus* or passive immunization with neutralizing sera provided immune protection against the skin infection of USA300. Our data illustrate for the first time that commensal *S. aureus* elicits both innate and adaptive immunity via glycerol fermentation and systemic antibody production, respectively, to fight off the skin infection of pathogenic MRSA.

## 1. Introduction

*Staphylococcus aureus* is a Gram-positive bacteria that colonizes the nasal cavity of 20–30% of the human population without causing any apparent disease [1]. Under certain circumstances, the bacteria can act as a pathogen to invade tissues and cause diseases. The factors determining the difference between its commensal and pathogenic states are still largely unknown. It has been reported that upregulation of *sdrC*, *fnbA*, *fhuD*, *sstD*, and *hla* genes occurs in *S. aureus* acting as an invasive pathogen compared to the commensals [2]. Results from a previous study revealed that the peptidoglycan layer of the staphylococcal cell wall binds to Toll-like receptor 2 (TLR2) on host antigen-presenting cells and induces a strong interleukin (IL)-10 response that downregulates the adaptive T-cell response [3]. This finding provides an explanation for the duality of interactions between *S. aureus* and host immunity by favoring nasal colonization as commensals instead of inducing staphylococcal diseases [4].

Mounting evidence has demonstrated that some human commensals not only directly benefit the host, but also play a crucial role in defense against pathogens [5]. Fermentation, one of the common activities that occurs between host and commensal bacteria, is one mechanism through which commensals inhibit pathogenic bacterial growth. Our previous studies demonstrated that human skin commensal bacteria, which can be defined as a part of skin innate immunity, inhibited the growth of USA300, a strain of community-acquired methicillin-resistant *S. aureus* (MRSA), by fermenting glycerol to release short-chain fatty acids (SCFAs) [6,7,8]. SCFAs can diffuse into the bacterial cell, reduce the intracellular pH of USA300, and eventually kill USA300. It has been documented that SCFAs in the skin play a vital role in influencing the composition of bacteria on normal human skin [9]. Human skin naturally synthesizes glycerol, which commensal bacteria use as a carbon source to produce SCFAs via fermentation [7]. Thus, fermentation in human skin may be a defense mechanism of the innate immunity, through which SCFAs are produced and distributed throughout all skin sites to regulate the composition of skin commensals and protect the human body from invading pathogens.

While current literature has provided information that several SCFAs have been detected in commensal fermentation in the gut, SCFA production in the glycerol fermentation pathway of commensal bacteria at other body sites has not been addressed [10]. Since *S. aureus* is a member of the human microbiome, the fermentation activities of commensal *S. aureus* and pathogenic USA300 were compared. The evolutionary reason of how *S. aureus* transitioned from a harmless commensal to an opportunistic pathogen remains a mystery. It has been proposed that all strains of MRSA stemmed from a single ancestral *S. aureus* strain that acquired *mecA*, but other studies show that some strains of MRSA are very divergent, suggesting that *mecA* has been transferred between *S. aureus* lineages [11]. In this study, we demonstrated that commensal *S. aureus*, but not USA300, was able to fermentatively metabolize glycerol to SCFAs. The deficiency of glycerol fermentation in USA300 may provide biological evidence for distinguishing commensal *S. aureus* from MRSA.

It has been reported that high-titer anti-*S. aureus* antibodies are stable for years in healthy individuals, and circulating anti-*S. aureus* serum antibodies in healthy individuals are functional based on their in vivo opsonophagocytic and neutralizing activities [12]. It has been illustrated that antibodies against protein components of *S. aureus*—such as surface adhesins, lipoproteins (Pbp2a), as well as secreted virulence factors including α-hemolysin [13]—are of benefit in warding off staphylococcal infection. The α-hemolysin encoded by the *hla* gene is crucial for *S. aureus* pneumonia, sepsis, and brain abscess. Vaccination with α-hemolysin was recently shown to protect mice against lethal *S. aureus* pneumonia [14]. As antibodies could offer protection against *S. aureus* infection, we speculate that natural antibodies in bloodstream provoked by commensal *S. aureus* are a key part of host adaptive immunity to prevent colonization of pathogenic *S. aureus*.

Current treatment options for MRSA skin and soft-tissue infection include the use of antibiotics and drainage of abscesses. Clindamycin or cotrimoxazole remains the antibiotics of choice for less serious, non-multiresistant MRSA infections [15]. Vancomycin or teicoplanin is used for more serious MRSA and multiresistant MRSA infections [15]. Some core problems complicating antibiotic therapy include a lack of selectivity, reduced susceptibility over time, and rapid emergence of antibiotic resistance. The problems with antibiotic treatments for MRSA infection call for exploration of other novel therapeutic options. Here, we demonstrate the probiotic activity of commensal *S. aureus*, which utilizes glycerol to yield antimicrobial SCFAs against MRSA. Furthermore, commensal *S. aureus* is able to produce neutralizing antibodies to α-hemolysin. In comparison to antibiotics, probiotic treatments or the use of neutralizing antibodies have a lower risk of inducing antibiotic-resistant microbes and have little or no disruption to other commensal bacteria. Our results here demonstrate for the first time that commensal *S. aureus* as a member of the skin microbiome can mediate both innate and adaptive immune responses to ward off the skin colonization of pathogenic MRSA.

## 2. Results

### 2.1. Interference of Commensal S. aureus with the Growth of USA300

**Figure 1:**
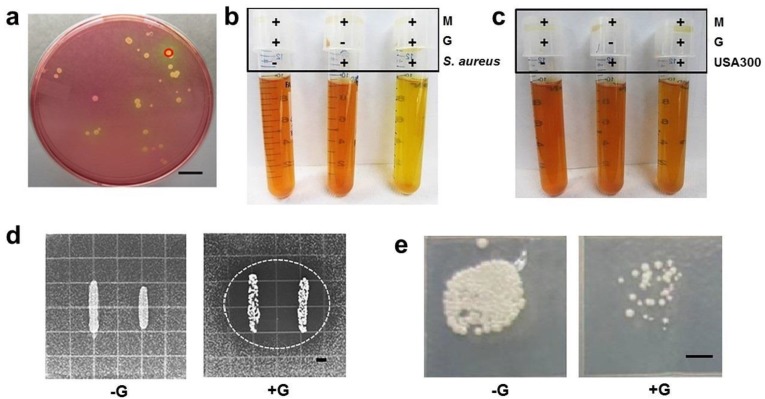
Differential activities of glycerol fermentation of commensal *Staphylococcus aureus* and USA300 and bacterial interference in the via glycerol fermentation. (**a**) A colony with a yellow zone (circle) was identified as commensal *S. aureus* by 16S rRNA sequencing; (**b**) isolated commensal *S. aureus* or (**c**) USA300 was incubated in rich media (M) with/without glycerol (G) for ten days. Rich media plus glycerol without *S. aureus* or USA300 were included as a control; (**d**) an overlay assay reveals a zone (circled) of inhibition in USA300 growth when commensal *S. aureus* and USA300 were grown with glycerol (+G). No inhibition zone developed when two bacteria were grown without glycerol (−G); (**e**) commensal *S. aureus* was co-cultured with USA300 in the presence (+G) or absence (−G) of glycerol. After a 4-day culture, media were spotted on benzylpenicillin-containing tryptic soy broth (TSB) plates overnight. Bars = 0.5 cm.

Skin bacteria were isolated from skin around the nose of a healthy male subject without infection. As shown in Figure 1a, a yellow colony in a mannitol salt agar plate was selected from a MSA plate for 16S ribosomal RNA (rRNA) sequencing. The 16S rRNA gene of this colony shares 97% identity with 16S rRNA gene in *S. aureus* NCTC8325. Bacteria isolated from this colony thus were assigned as commensal *S. aureus* since they were derived from normal microflora in humans. To test the fermentative capabilities, commensal *S. aureus* and USA300 were cultured in rich media under anaerobic conditions in the presence of glycerol for ten days. Rich media with glycerol and rich media with commensal *S. aureus* or USA300 alone served as controls. As shown in Figure 1b, the pH values of media with glycerol, commensal *S. aureus*, and glycerol plus commensal *S. aureus* were 7.5 ± 0.2, 7.3 ± 0.3, and 6.5 ± 0.2, respectively. The media in the culture of commensal *S. aureus* with glycerol turned yellow after four days of incubation, while the media in the culture of USA300 maintained its original color for the ten days (Figure 1c), indicating that commensal *S. aureus* bacteria, but not USA300, had a capability for glycerol fermentation.

Two sets of experiments were conducted to examine whether the glycerol fermentation of commensal *S. aureus* hindered the growth of USA300. In the first set of experiments, we used an overlay assay [7] to detect the bacterial interference on agar plates supplemented with/without glycerol (20 g/L). As shown in Figure 1d, only commensal *S. aureus* grown with glycerol formed visible inhibitory zones against USA300. No inhibitory zone was observed when commensal *S. aureus* and USA300 were grown in the absence of glycerol. In the second set of experiments, commensal *S. aureus* was co-cultured with USA300 in the presence or absence of glycerol. To establish a USA300-selective plate, media from the co-culture of commensal *S. aureus* and USA300 was spotted on a rich medium plate supplemented with benzylpenicillin. We found that benzylpenicillin at a concentration of 32 µL/mL can completely kill commensal *S. aureus* without affecting the growth of USA300 (Figure S1). Four days after the co-culture of commensal *S. aureus* and USA300 with/without glycerol, media were spotted on a USA300-selective plate. After co-culture of commensal *S. aureus*/USA300 in the absence of glycerol, USA300 grew high-density colonies on a plate. However, when glycerol was present in the co-culture, only a few USA300 colonies were observed (Figure 1e). These findings suggest that commensal *S. aureus* mediated glycerol fermentation to interfere with the growth of USA300. To examine whether other commensal *S. aureus* isolates exhibit anti-USA300 activities similar to one isolate observed in Figure 1e, three different colonies of commensal *S. aureus* isolated from human skin were selected for co-culture with USA300 in the presence or absence of glycerol. As shown in Figure S2, all three commensal *S. aureus* isolates can interfere with the growth of USA300 in the presence of glycerol, demonstrating the generalizability of commensal *S. aureus* against USA300.

### 2.2. Differential Expression of Enzymes in the Pathway of Glycerol Fermentation

**Figure 2:**
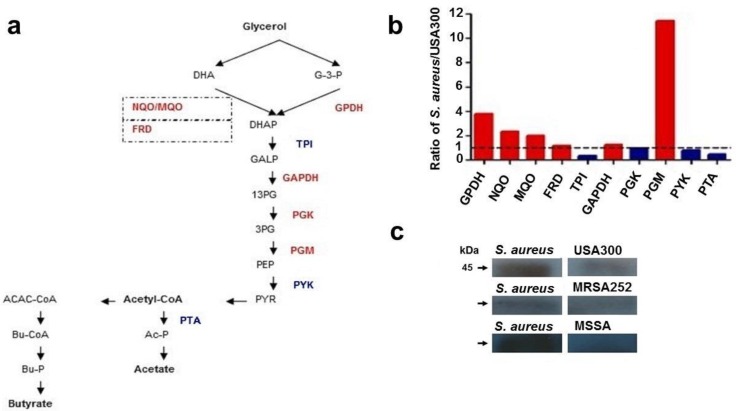
Mass spectrometric quantification of enzymes involved in glycerol fermentation. (**a**) Enzymes with higher (red) or lower (blue) abundance in commensal *S. aureus* than USA300 are denoted in the pathway of glycerol fermentation to butyrate or acetate. Enzymes involved in the conversion of dihydroxyacetone (DHA) to DHA phosphate (DHAP) are marked with squares with dashed lines; (**b**) relative abundance of (co-)enzymes in commensal *S. aureus* to those in USA300 (ratio of *S. aureus*/USA300) was calculated from data obtained by label-free quantification of LC/MS/MS. A dotted line is drawn at a ratio of 1; (**c**) the expression of glycerol-3-phosphate dehydrogenase (GPDH) in USA300, MRSA252, MSSA, and commensal *S. aureus* derived from three colonies isolated from a male subject was detected by Western blotting with anti-GPDH antibodies. The observed molecule weight (kDa) of GPDH is indicated. GALP, glyceraldehyde 3-phosphate; 13PG, 1,3-diphosphoglycerate; 3GP, 3-glycerophosphate; PEP, phosphoenolpyruvate; PYR, pyruvate; ACAC-CoA, acetoacetyl-CoA; Ac-P, acetyl phosphate; Bu-CoA, butyryl-CoA; Bu-P, butyryl phosphate. Abbreviations for names of enzymes are listed in Table S1.

Glycerol fermentation, an anaerobic process, requires many enzymes which degrade glycerol to SCFAs. A method of mass spectrometric label-free protein quantification [16] was used to investigate whether the distinction in the fermentative capabilities of commensal *S. aureus* and USA300 is due to the differential expression of enzymes involved in the glycerol fermentation. The abundance of ten enzymes (Figure 2a) that convert glycerol to butyrate or acetate was quantified. Relative expression ratios (commensal *S. aureus* to USA300) of enzymes that fermentatively metabolize the glycerol to either butyrate or acetate are presented in Figure 2b. Commensal *S. aureus* has higher levels of glycerol-3-phosphate dehydrogenase (GPDH), NAD(P)H:quinone (NQO), malate-quinone oxidoreductase (MQO), succinate dehydrogenase and fumarate reductase iron–sulfur protein (FRD), glyceraldehyde-3-phosphate dehydrogenase (GAPDH), and phosphoglycerate mutase (PGM) than USA300, but lower levels of triosephosphate isomerase (TPI), phosphoglycerate kinase (PGK), pyruvate kinase (PYK), and phosphate acetyltransferase (PTA). Since GPDH catalyzes the oxidation of glycerol-3-phosphate (G-3-P) to dihydroxyacetone phosphate (DHAP) in the upstream pathway of glycerol fermentation, the expression of GPDH in various *S. aureus* strains was measured by Western blot using anti-GPDH antibodies. To investigate whether the differential abundance of GPDH between commensal and pathogenic *S. aureus* is commonly detectable, we performed the Western blot analysis to quantitatively compare the levels of GPDH in commensal *S. aureus*, MRSA252, a hospital-acquired MRSA strain, and methicillin-susceptible *S. aureus* (MSSA), an invasive *S. aureus* strain. As shown in Figure 2c, expression level of GPDH in USA300, MRSA252, or MSSA was much lower than that in commensal *S. aureus*. Consistently, USA300 (Figure 1c), MRSA252, or MSSA, but not commensal *S. aureus*, developed a significant deficiency in the glycerol fermentation. The results indicate that, unlike commensal *S. aureus*, several pathogenic *S. aureus* bacteria are incapable of fermenting glycerol. The results also suggest that insufficient expression of GPDH in the upstream pathway of fermentation may be a cause for the failure of glycerol fermentation in pathogenic *S. aureus*.

### 2.3. SCFAs in Fermentation Metabolites of Commensal S. aureus and Suppression of USA300 Growth In Vivo

**Figure 3:**
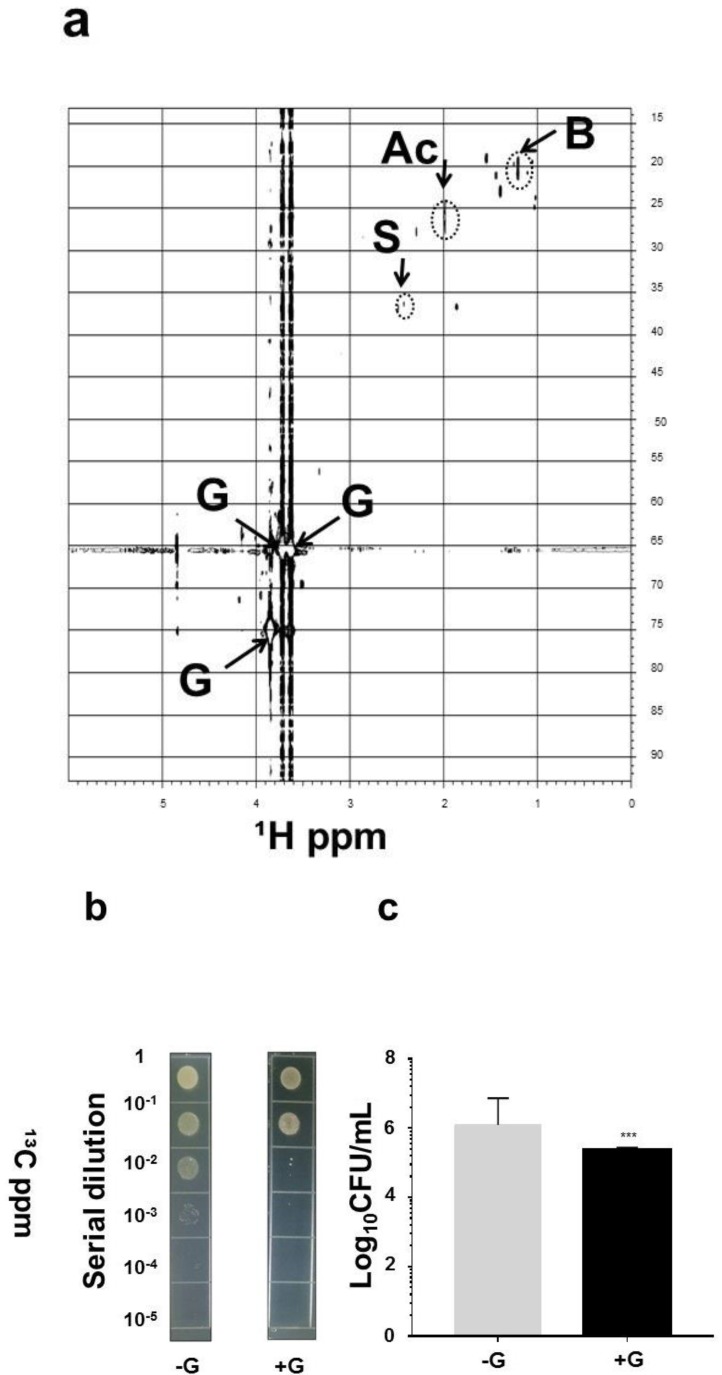
SCFAs produced by *S. aureus* and in vivo interference of *S. aureus* with the growth of USA300 in the presence of glycerol. Fermented media of commensal *S. aureus* were centrifuged and passed through a 0.2 μm filter. (**a**) The chief SCFAs in the fermented media after incubation with ^13^C_3_-glycerol for four days are displayed in a 2-D ^1^H-^13^C HSQC nuclear magnetic resonance (NMR) spectrum (600 MHz). Besides glycerol (G), three SCFAs (acetic acid (Ac), butyric acid (B), and succinic acid (S)) were detected in the ferments of commensal *S. aureus*; (**b**) a volume of 10 µL of PBS of commensal *S. aureus* (10^8^ CFU) and USA300 (10^8^ CFU) with (+G)/without (−G) 2% glycerol were applied on the skin wounds (1 cm) of ICR mice for 3 days. Bacterial CFUs in the skin wounds were enumerated by plating serial dilutions (1:10^1^ to 1:10^5^) of the homogenate on a benzylpenicillin (32 µg/mL)-containing TSB plate; (**c**) the number of viable bacterial colonies is expressed as log_10_ CFU/mL. *** *p* < 0.001 (two-tailed *t*-tests). Data are the mean ± SD of three individual experiments.

To identify the SCFAs in the products of glycerol fermentation, commensal *S. aureus* was incubated in rich media with ^13^C_3_-glycerol for four days. Supernatants of fermentation in 10% D_2_O were subjected to 1-D (Figure S3) and two-dimensional (2-D) ^13^C and ^1^H (Figure 3a) nuclear magnetic resonance (NMR) analysis. Acetate, butyrate, and succinate are three major SCFAs produced by glycerol fermentation of commensal *S. aureus*. Results in our previous publication [17] have demonstrated that the minimum bactericidal concentration (MBC) values (>1 log_10_ inhibition) of butyric acid and acetic acid for USA300 were 10 mM and the concentration of both acids for complete inhibition was 50 mM, suggesting that SCFAs produced by glycerol fermentation of commensal *S. aureus* are able to impede the growth of USA300. To validate whether glycerol fermentation of commensal *S. aureus* can hinder the growth of USA300 in vivo, commensal *S. aureus* and USA300 with/without 2% glycerol for 3 days were applied onto the wounded skin of Institute Cancer Research (ICR) mice. The skin wounds in mice present an animal model of external traumatic wound infections of MRSA in humans [18]. As shown in Figure 3b,c, the number ((2.3 ± 1.9) × 10^5^ CFU) of USA300 bacteria in the bacteria-inoculated wound with glycerol was significantly less than that ((2.7 ± 1.4) × 10^6^ CFU) in the bacteria-inoculated wound without glycerol. The result demonstrates that the commensal *S. aureus* possesses probiotic activity against the USA300 skin infection.

### 2.4. Protection of Skin Infection of USA300 by Antibodies to α-Hemolysin of Commensal S. aureus

**Figure 4:**
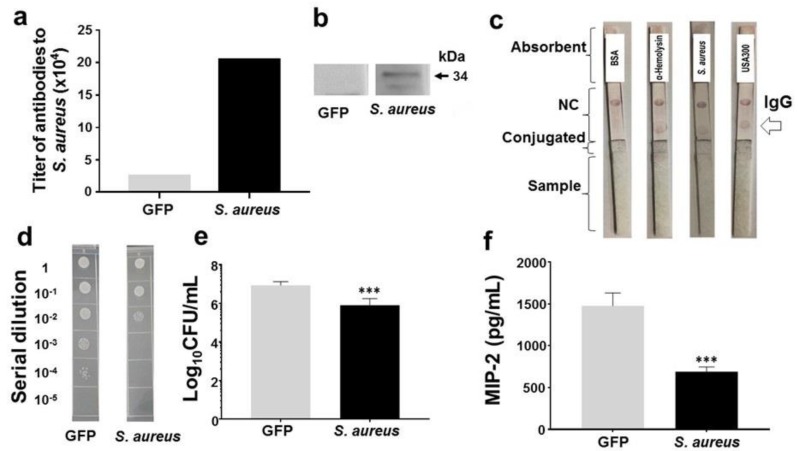
The production of antibodies to α-hemolysin and USA300 by vaccination of commensal *S. aureus* and protection against USA300. ICR mice subcutaneously vaccinated with lysates of commensal *S. aureus* (but not those vaccinate with recombinant GFP) produced detectable antibodies (IgG) to *S. aureus* (**a**) or α-hemolysin (**b**) in quantitative OD_570-450_ measurement and Western blot analysis, respectively; (**c**) the production of antibodies to *S. aureus* or α-hemolysin and their cross-reactivity with USA300 was detected in test strips with NC membranes spotted with BSA, α-hemolysin, or lysates of *S. aureus* or USA300 (lower position) and rabbit anti-mouse IgG secondary antibody (upper position); (**d**) a 1 cm wound was made on the dorsal skin of vaccinated mice, followed by applying USA300 for 3 days. Bacterial CFUs in the skin wounds were enumerated by plating serial dilutions of the homogenate on a plate. The number (log_10_ CFU/mL) of USA300 (**e**) and the level of pro-inflammatory MIP-2 cytokine (**f**) were quantified. *** *p* < 0.001 (two-tailed *t*-tests). Data are the mean ± SD of three separate experiments.

By the method of mass spectrometric label-free protein quantification, we found that commensal *S. aureus* expresses α-hemolysin (accession number: Q6SV31). It has been documented that active or passive immunization of α-hemolysin decreases the severity of skin infection of USA300 in mice [19]. Thus, we tested whether the α-hemolysin of commensal *S. aureus* is immunogenic and whether antibodies produced by immunization of commensal *S. aureus* lysates or α-hemolysin provide protection against the skin infection of USA300. The α-hemolysin of commensal *S. aureus* was cloned and expressed. The recombinant α-hemolysin was purified and confirmed by the sodium dodecyl sulfate-polyacrylamide gel electrophoresis (SDS-PAGE) gels staining with Coomassie blue (Supplementary Materials). The gene sequences of α-hemolysin of commensal *S. aureus* (Figure S4) share 99% identity with those (accession number: SAUSA300_1058 (ABD20868)) of α-hemolysin of USA300. Subcutaneous immunization of ICR mice with lysates of commensal *S. aureus* (but not recombinant GFP) with 2% alhydrogel elicited detectable antibodies (immunoglobulin G (IgG)) to commensal *S. aureus* in ELISA (Figure 4a) and α-hemolysin in Western blot (Figure 4b), demonstrating the immunogenicity of α-hemolysin of commensal *S. aureus*. The immunochromatographic test strips were fabricated and spotted with bovine serum albumin (BSA), α-hemolysin, or lysates of *S. aureus* or USA300. The sera of mice immunized with lysates of commensal *S. aureus* were added onto the test strips. As shown in Figure 4c, α-hemolysin and lysates of commensal *S. aureus* or USA300, but not BSA, can be recognized by the antibodies in sera, suggesting that immunization of lysates of commensal *S. aureus* provoked antibodies that can cross-react with USA300. To determine if the antibody cross-reactivity offers a protection against USA300 infection, skin wounds in mice immunized with lysates of commensal *S. aureus* or GFP were inoculated with USA300 bacteria. The wounds were homogenized to measure the intensity of USA300 colonization. The USA300 counts in wounds in mice immunized with lysates of commensal *S. aureus* or GFP were 5.9 ± 0.2 and 6.9 ± 0.3 log_10_ CFU/mL, respectively (Figure 4d,e). It is known the binding of MRSA to TLR2 provokes secretion of pro-inflammatory cytokine of human interleukin (IL)-8, a counterpart of mouse macrophage inflammatory protein 2 (MIP-2) [20]. As shown in Figure 4f, in GFP-immunized mice, a significant increase in the level of MIP-2 in skin wounds was observed 3 days after USA300 inoculation, while *S. aureus*-immunized mice demonstrated 53% less induction.

**Figure 5:**
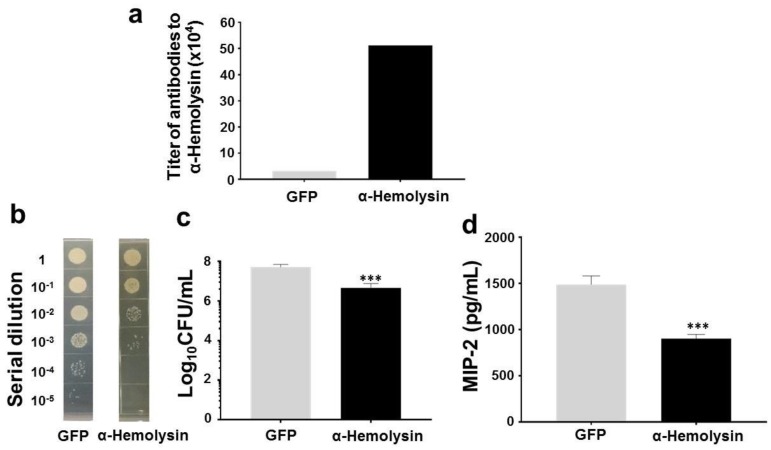
Protection against USA300 by vaccination of commensal *S. aureus* α-hemolysin. ICR mice subcutaneously vaccinated with recombinant α-hemolysin or GFP (50 µg). (**a**) The antibodies (IgG) to α-hemolysin were detected in quantitative OD_570-450_ measurement; (**b**) USA300 (10^8^ CFU) were applied onto a skin wound of vaccinated mice for 3 days. Bacterial CFUs in the skin wounds were enumerated by plating serial dilutions (1:10^1^–1:10^5^) of the homogenate on a plate; The number (log_10_ CFU/mL) of USA300 (**c**) and the level of pro-inflammatory MIP-2 cytokine (**d**) were quantified. *** *p* < 0.001 (two-tailed *t*-tests). Data shown represent the mean ± SD of experiments performed in triplicate.

To investigate whether immunization of α-hemolysin of commensal *S. aureus* prompts protective immune responses against USA300, skin wounds in GFP- or α-hemolysin-immunized mice were inoculated with USA300 bacteria. In comparison with GFP-immunized mice, α-hemolysin-immunized mice had markedly less USA300 and a lower MIP-2 level in skin wounds (Figure 5). Results above suggest that immunization of commensal *S. aureus* or α-hemolysin significantly decreased the growth of USA300 and level of MIP-2 in the skin wounds. Furthermore, the passive neutralization of USA300 skin infection by antisera to *S. aureus* or α-hemolysin is demonstrated in Figure S5.

## 3. Discussion

Genomic analysis of *S. aureus* strains has helped to identify a number of virulence factors [21]. Community-associated MRSA is characterized by a short SCCmec and the presence of a Panton-Valentine leucocidin (PVL) locus. Mass spectrometric analysis showed many proteins associated with the pathways of peptidoglycan biosynthesis and the pantothenate and CoA biosynthesis were upregulated in both oxacillin-treated MRSA and MSSA, and a number of proteins involved in the pathways of energy metabolism were upregulated exclusively in oxacillin-treated MSSA [22]. In this study, we found that the abundance of six enzymes, including GPDH and PGM, involved in the pathway of glycerol fermentation in commensal *S. aureus* was higher than that in USA300, while the levels of four enzymes, including TPI and PTA, in commensal *S. aureus* were lower than those in USA300 (Table S1). Although we here define those *S. aureus* bacteria isolated from human skin around nose as commensal *S. aureus*, it has been reported that the nasal carriage can be a source of *S. aureus* bacteremia [23]. Therefore, genomic analysis and antibiotic susceptibility testing for further characterization of these *S. aureus* isolates may be necessary to determine their possible virulence.

The human skin has a self-sterilizing activity which has been ascribed to various factors such as low pH or some antimicrobial agents, including SCFAs. Numerous SCFAs, although at relatively low concentrations, are commonly detectable in the skin and in the secretions of skin glands, such as the sweat. Although it is still not clear at what abundance SCFAs can be produced by glycerol fermentation of skin commensal *S. aureus*, human sweat contains 0.0096% acetate [24]. The SCFAs produced by intestinal microbes in the human colon can reach a high level (20–140 mM) [24] that can effectively kill local pathogens. The concentrations of SCFAs in peripheral circulation are generally low, ranging from 3 to 7 µM [25]. Results from our pervious publication [25] have demonstrated that propionic acid produced by glycerol fermentation of *Propionibacterium acnes* (*P. acnes*) and a propionic acid derivative can effectively suppress the growth of USA300. In the future, determination of the amount of SCFAs produced from bacterial fermentation in skin will provide valuable information on the formulation and dosage schedule of SCFAs and/or their analogues as new antimicrobials for treatment of MRSA skin infections. As shown in Figure 2c, the amount of GPDH in USA300, MRSA252, or MSSA was much lower than that in commensal *S. aureus*. Although several SCFAs produced by fermentation of skin commensal bacteria [17,26] can inhibit the growth of USA300, future works will investigate the antimicrobial activities of SCFAs against other pathogenic *S. aureus* strains, including MRSA and MSSA. Although mouse skin with wounds [18] was used as an in vivo model to study the interference of *S. aureus* fermentation with the growth of USA300 (Figure 3b,c), future studies will long-term colonize mouse skin with human commensal *S. aureus* [27] before inoculation with USA300 to examine how commensal bacteria modulate skin immunity against USA300.

The antibodies to α-hemolysin are detectable in the circulating blood in humans [28]. Although immunization of mice with recombinant α-hemolysin yielded high antibody titers of more than 1:512,000, which provided great protection against skin infection of USA300 (Figure 5), we do not know how high titers of antibodies to α-hemolysin in humans are sufficient to prevent the invasion of MRSA. The antibodies against 11 *S. aureus* antigens, including α-hemolysin, have been detected in human blood [29]. However, the high frequency of recurring MRSA strains, despite high titers of specific antibodies, denotes that traditional adaptive immunity imparts incomplete protection [30]. The *S. aureus* α-hemolysin has been selected as a protein target for active immunization in clinical studies for evaluating the efficacy of anti-α-hemolysin antibodies in nosocomial pneumonia [31]. The disappointing results of human trials have raised the question of whether other toxins, such as phenol-soluble modulins (PSMs) [32], contribute to the virulence of *S. aureus* and whether pre-existing antibodies to α-hemolysin affects the efficacy of vaccines against *S. aureus*. Moreover, the effect of bacteria in the skin microbiome, including commensal *S. aureus*, on α-hemolysin vaccination remains unclear. After binding to TLR2, *S. aureus* has been shown to provoke secretion of cytokines (tumor necrosis factor (TNF)-α, IL-1β, IL-10, IL-12, and IL-8) from macrophages [33]. Macrophages play a key role in mediating neutrophil recruitment during MRSA skin infection [34]. IL-8, or mouse MIP-2, is a strong chemoattractant for neutrophils. Although our results demonstrate that passive or active immunization of *S. aureus* lysates or α-hemolysin exerted anti-inflammatory activity via downregulation of USA300-induced MIP-2 cytokines (Figure 4f, Figure 5d, and Figure S5c), it is worth determining if downregulation of MIP-2 attenuates the recruitment of phagocytes to the infectious site of MRSA. Circulating antibodies to α-hemolysin in human bloodstream may bind α-hemolysin secreted from both commensal *S. aureus* and invasive USA300. As shown in Figure 4d,e, immunization of commensal *S. aureus* or α-hemolysin significantly reduced the colonization of USA300 in mouse ear, inferring that α-hemolysin immunization may disarm USA300 that could be eliminated locally and naturally by host immune system. Literature has shown that butyric acid produced by fermentation of commensal bacteria can induce the differentiation of regulatory T (Treg) cells [35], which promote the homeostasis of commensal bacteria without elimination by host immune systems [36].

*S. aureus* is commonly viewed as bacteria prone to solely inducing staphylococcal diseases in hospitals. However, the evidence from this study demonstrates that these bacteria may actually serve as a beneficial microbe in humans. The commensal *S. aureus* in skin can induce both innate immunity via production of SCFAs and adaptive immunity via production of antibodies to α-hemolysin. The new modalities derived from fermentation or vaccination of commensal *S. aureus* thereby benefit the entire community of patients with MRSA infections, consisting of over 126,000 patients per year in US [37]. In summary, we demonstrate for the first time that commensal *S. aureus*, but not USA300, can ferment glycerol to produce SCFAs. The systemic antibodies to commensal bacteria were abundantly present in humans and remained fairly stable over years [12]. They may play a central role in maintaining balance of the human microbiome and fighting systemic dissemination of pathogens [38]. We here demonstrate that the commensal *S. aureus* can generate antibodies to α-hemolysin against the skin infection of USA300. From the evolutionary point of view, commensal *S. aureus* bacteria may provide benefits to the host by maintaining their probiotic innate activity, which has been not evolutionarily conserved in MRSA. Existence of circulating antibodies to *S. aureus* α-hemolysin may be a host’s evolutionary strategy to prevent colonization of MRSA.

## 4. Materials and Methods

### 4.1. Ethics Statement

This study was carried out in strict accordance with an approved Institutional Animal Care and Use Committee (IACUC) protocol at National Central University (NCU), Taiwan. The Institutional Review Board (IRB) at University of California, San Diego (UCSD) approved the consent procedure and bacterial sampling under an approved protocol (No. 141735, 9 December 2014). The written consents from all participants were obtained before conducting bacterial sampling.

### 4.2. Bacterial Culture, Identification, Glycerol Fermentation, and Anti-USA300 Overlay Assays

Culture and identification of bacteria (USA300 [39], MRSA252, invasive methicillin-susceptible *S. aureus* (MSSA) (ATCC29213), or commensal *S. aureus*) are described in Supplementary Materials. To induce fermentation, bacteria (10^5^ colony-forming unit (CFU)/mL) were incubated in rich medium (10 g/L yeast extract (Biokar Diagnostics, Beauvais, France), 5 g/L tryptic soy broth (TSB), 2.5 g/L K_2_HPO_4_ and 1.5 g/L KH_2_PO_4_) in the absence and presence of 20 g/L (2%) glycerol under anaerobic conditions using Gas-Pak (BD) at 30 °C for ten days. Rich medium plus 20 g/L glycerol without bacteria was included as a control. The 0.001% (*w*/*v*) phenol red (Sigma, St. Louis, MO, USA) in rich medium with 20 g/L glycerol served as an indicator, converting from red-orange to yellow when fermentation occurred. SCFA identification by nuclear magnetic resonance (NMR) analysis and enumeration of USA300 by overlay assays and co-culture of USA300 with commensal *S. aureus* are described in detail in Supplementary Materials.

### 4.3. Mass Spectrometric Label-Free Protein Quantification and Western Blot

The one-dimensional (1-D) liquid chromatography-tandem mass spectrometry (LC-MS/MS) data has been submitted to Integrated Proteomics Pipelines (IP2)/Census for peptide/proteins identification and label-free quantification analysis [16], which appears in Supplementary Materials online. Detailed protocols for Western blot analysis can be found in Supplementary Materials online.

### 4.4. In Vivo Effects of S. aureus Glycerol Fermentation on Skin Infection of USA300

The Institute Cancer Research (ICR) mice (8–12-month-old females; National Laboratory Animal Center, Taipei, Taiwan) were anesthetized by isoflurane. A 1 cm wound was made on the dorsal skin following shaving with electrical clippers. Following skin wounding, we applied 10 µL of phosphate buffered saline (PBS) of commensal *S. aureus* (10^8^ CFU) and USA300 (10^8^ CFU) with/without 2% glycerol for 3 days to the wounded areas. To measure the extent of wound closure, the wounded skin was covered with a transparent parafilm and the outline of the wound margin was traced onto the parafilm. The lesion size (cm^2^) was recorded daily and quantified by ImageJ software 1.50b (National Institutes of Health (NIH), Bethesda, MD, USA). To count the bacterial numbers in infected skin, the skin was excised and homogenized in 200 µL of sterile PBS with a tissue grinder 3 days after bacterial application. The CFUs of bacteria in the skin were enumerated by plating serial dilutions (1:10^1^ to 1:10^5^) of the homogenate on a benzylpenicillin (32 µg/mL)-containing TSB plate. Bacterial colonies on the plates were formed after overnight incubation at 37 °C. The bacterial numbers (CFUs/mL) of excised skin were calculated. The pro-inflammatory MIP-2 cytokine was determined by sandwich enzyme-linked immunosorbent assay (ELISA) using a Quantikine mouse MIP-2 set (R&D Systems, Minneapolis, MN, USA).

### 4.5. Vaccination, Antibody Detection, and Protection against USA300

Female ICR mice approximately 8–12 weeks old were used for vaccination. Lysates of commensal *S. aureus*, recombinant green fluorescent protein (GFP), or α-hemolysin (50 µg) was dissolved in PBS (100 µL) and mixed with an equal volume (100 µL) of 2% alhydrogel (InvivoGen, San Diego, CA, USA). The method for molecular cloning and expression of *S. aureus* α-hemolysin was posted in Supplementary Materials. For the first vaccination, 50 µg of *S. aureus* lysates or recombinant proteins in alhydrogel was injected subcutaneously into the dorsal skin. Two weeks later, the same amounts of lysates or proteins in alhydrogel were injected for second boost. One week after the second boost, serum containing IgG was detected by a microplate reader at OD_570_/OD_450_ and test strips (Supplementary Materials). A 1 cm wound was made on the dorsal skin of immunized mice before applying USA300 (10^8^ CFU in 10 µL of PBS) for 3 days. The number (CFUs) of USA300 and the level of pro-inflammatory MIP-2 cytokine were quantified as described above.

### 4.6. Statistical Analysis

The two-tailed *t*-test was used to compare values of means and define significances between groups. Five mice per group per experiment were performed for in vivo experiments. Data denote the mean ± standard deviation (SD) from three separate experiments. The *p*-values of <0.01 (**), and <0.001 (***) were accepted for statistical significance.

## Supplementary Materials

Supplementary Materials can be found at http://www.mdpi.com/1422-0067/19/5/1290/s1.
