# Supplementary material for: Commensal Staphylococcus aureus Provokes Immunity to Protect against Skin Infection of Methicillin-Resistant Staphylococcus aureus
Source: Int J Mol Sci. 2018 Apr 25;19(5):1290. doi: 10.3390/ijms19051290 (PMC5983722; doi:10.3390/ijms19051290)
Supplement: Supplementary file 1 [file ijms-19-01290-s001.pdf]

Supplementary Materials: Commensal *Staphylococcus aureus* Provokes Immunity to Protect against Skin Infection of Methicillin-Resistant *Staphylococcus aureus*

John-Jackson Yang, Ting-Wei Chang, Yong Jiang, Hsin-Jou Kao, Bin-Hao Chiou, Ming-Shan Kao and Chun-Ming Huang

1. Supplementary Information

1.1. Passive Neutralization of USA300 Skin Infection by Antisera to S. aureus or α-Hemolysin

To evaluate whether serum of mice immunized with lysates of commensal *S. aureus* or α-hemolysin can hinder the growth of USA300 in skin wounds and neutralize the production of MIP-2 cytokines, USA300 bacteria were pre-treated with anti-serum to *S. aureus* or α-hemolysin for 1 h prior to being added onto skin wounds. The CFU counting on agar plates showed that 1 h treatment with antisera did not impair the growth of USA300. USA300 pre-treated with anti-GFP serum caused 6.8 ± 0.3 $\log_{10}$ CFU/mL of USA300 growth in skin wounds, while pre-treatment with serum to *S. aureus* or α-hemolysin dramatically reduced the growth of USA300 to 5.5 ± 0.1 or 5.6 ± 0.2 $\log_{10}$ CFU/mL, respectively (Figure S5a,b). Furthermore, pre-treatment with serum to *S. aureus* or α-hemolysin significantly lowered the levels of MIP-2 in skin wounds (Figure S5c). This data suggests that mice immunized with commensal *S. aureus* produce neutralizing antibodies that can fight off the skin infection of USA300.

1.2. Materials and Methods

1.2.1. Bacterial Culture and Identification

Bacteria including USA300 [1], MRSA252, invasive MSSA (ATCC29213), or commensal *S. aureus* were cultured in 3% tryptic soy broth (TSB) (Sigma, St. Louis, MO, USA) agar plates overnight at 37 °C. Commensal *S. aureus* bacteria were isolated by moving a sterile inoculating loop (Thermo Fisher Scientific, San Diego, CA, USA) over the area of skin around the nose of male subjects and immediately inoculated onto a MSA plate (BD, Sparks, MD, USA) overnight. Bacterial colonies selected from agar plates were subsequently cultured in TSB [2]. Overnight cultures were diluted 1:100 and cultured to an absorbance at 600 nm [optical $density_{600}$] = 1.0. Bacteria were harvested by centrifugation at 5000× *g* for 10 min, washed with phosphate buffered saline (PBS), and suspended in PBS for further experiments. Sequence analysis of 16S rRNA genes [1] was utilized for bacterial identification. A single colony of bacteria, which created a yellow colony on MSA plates, was picked up by sterile toothpicks and boiled at 100 °C for DNA extraction. The polymerase chain reaction (PCR) with 16S rRNA 27F and 534R primers in addition to sequencing of PCR products was performed as previously described [3]. The 16S rRNA gene sequences were analyzed using the basic local alignment search tool (BLASTn, National Library of Medicine 8600 Rockville Pike, Bethesda, MD, USA).

1.2.2. Anti-USA300 Overlay Assays and Bacterial Co-Culture

Commensal *S. aureus* bacteria were mixed with 1.5% molten (*w*/*v*) agar (Oxoid. Ltd., London, UK) with/without glycerol (20 g/L) in rich media. Agar was cooled to 45 °C before bacteria were added to obtain a concentration of 10^7 CFU (in 20 mL). The bacterial suspension/agar was poured into plates to produce a homogeneous lawn of commensal *S. aureus*. The USA300 bacteria (10^5 CFU in 20 μL) were inoculated in two 2 cm streaks on top of the lawn of commensal *S. aureus*, and then

cultured under anaerobic conditions at 37 °C for two days. For bacterial co-culture, commensal *S. aureus* (10^7 CFU/mL) was co-incubated with USA300 (10^5 CFU/mL) in rich media in the absence and presence of 20 g/L glycerol under anaerobic conditions at 30 °C for four days. Media (5 μL) containing bacteria were spotted on rich medium (1.5%) agar plates supplemented with 8, 16, 32, or 64 μL/mL benzylpenicillin (Sigma) for overnight culture.

1.2.3. Mass Spectrometric Label-Free Protein Quantification

Bacteria (10^5 CFU/mL) were cultured in rich media overnight. After centrifugation at 5000× *g* for 30 min, the bacterial pellets (100 μg) in 2% SDS buffer were digested using filter-aided sample preparation (FASP) method [4]. Protein reduction and alkylation were carried out in presence of 8 M urea on filter by adding dithiothreitol (DTT) (10 mM) and incubating at 32 °C for 60 min. Iodoacetamide (30 mM) was added for alkylation at 20 min at room temperature in the dark. Mass spectrometry grade trypsin (Promega, Madison, WI, USA) (1:20 ratio) was used for overnight digestion at 32 °C. After digestion, formic acid (1%) was added to samples, followed by desalting using a C18 TopTip (PolyLC Inc., Columbia, MD, USA). The LC-MS/MS analysis was carried by on-line analysis of peptides by high-resolution, high-accuracy LC-MS/MS, consisting of an EASY-nLC 1000 high performance liquid chromatography (HPLC) Acclaim PepMap peptide trap, a 25-cm 2 μm Easy-Spray C18 column, Easy Spray Source, and a Q Exactive Plus mass spectrometer (Thermo Fisher Scientific). A 180-min gradient consisting of 5–16%B (100% acetonitrile) in 140 min, 16–28% in 70 min, 28–38% in 10 min, and 38–85% in 5 min was used to separate the peptides. The total LC time was 240 min. The Q Exactive Plus was set to scan precursors at 70,000 resolution followed by data-dependent MS/MS at 17,500 resolution of the top 12 precursors. Raw LC-MS/MS data was then submitted to Integrated Proteomics Pipelines (IP2)/Census for peptide/proteins identification and label-free quantification analysis [5]. The LC-MS/MS raw data were submitted to IP2 Version IP2 1.01 (Integrated Proteomics Applications, Inc., San Diego, CA, USA) with ProLucid algorithm as the search program for peptide/protein identification. ProLucid search parameters were set up to search the SwissProt_Aureus_ET3_COL_JH1_MRSA252 (version 01-01-2014) fast database including reversed protein sequences using trypsin with the allowance of up to two missed cleavages, semi-tryptic search and precursor mass tolerance of 50 ppm. Differential search included 16 Da for methionine oxidation and 57 Da for that of cysteine to account for carboxyamidomethylation of cysteines. The search results were viewed, sorted, filtered, and statistically analyzed by using DTASelectwith best peptide delta mass threshold (-tDM) of 10 ppm, best peptide FP threshold (-tfp) of 0.01 and protein false discovery rate (FDR) of less than 2.5%. Differential label-free proteomics data analysis was done by IP2-Census using two technical replicates datasets per sample [6].

1.2.4. Identification of SCFAs by NMR Analysis

Commensal *S. aureus* (10^5 CFU/mL) was incubated in phenol red-free rich media with $^{13}C_3$-glycerol (20 g/L) (Cambridge Isotope Laboratories, Andover, MA, USA) for four days. After removing bacteria by centrifugation at 5000× *g* for 30 min, fermented media were passed through 0.2 μm pore-size filters. SCFAs in the bacteria-free media were analyzed by NMR spectrometers. The 1-D NMR spectra were measured on a JEOL-ECS NMR spectrometer operating at a resonance frequency of 400 MHz with a repetition delay of 3 s for both ^1H and ^{13}C. The 2-D ^1H-^{13}C heteronuclear single quantum correlation (HSQC) NMR spectra were acquired on a Bruker Avance 600 MHz NMR spectrometer with a triple resonance inverse (TCI) cryo-probe and recorded as 2048 × 256 complex points with 32 scans and 1 s repetition time. Newly appearing peaks belonged to the intermediate or final metabolites resulting from $^{13}C_3$-glycerol fermentation by bacteria [3].

1.2.5. Molecular Cloning and Expression of Recombinant α-Hemolysin

A PCR product encoding α-hemolysin of commensal *S. aureus* (accession number: Q6SV31) was generated using gene-specific primers. The forward PCR primer with EcoRI (5′-GGGGGGAATTCCATGAAAACACGTATAGT-3′) and reverse PCR primer with XhoI

(5′-GGGGGCTCGAGATTTGTCATTTCTTCTTT-3′). PCR was performed using the forward and reverse primers and genomic DNA of commensal *S. aureus* as a template. The amplified fragment was inserted into a pET21b (Ampr T7 expression region N-terminal T7 Tag C-terminal His Tag) expression plasmid. Competent cells (*Escherichia coli* (*E. coli*), BL21 (DE3), Invitrogen, Carlsbad, CA, USA) were transformed with this plasmid, selected on Luria–Bertani (LB) plates containing ampicillin (50 μg/mL) and an aliquot of the overnight culture was diluted 1:20 with LB medium and incubated at 37 ℃ until reaching OD_{600} = 0.7. Isopropyl-β-D-thiogalactoside (IPTG) (1 mM) was added into culture for 4 h to induce protein synthesis. The expressed α-hemolysin possessing 6× His tag was purified in denaturing conditions with a TALON Express Purification Kit (Clontech Laboratories, Inc., Mountain View, CA, USA). Subsequently, gel staining with Coomassie blue was performed to detect the expression of α-hemolysin.

1.2.6. Western Blot

Bacterial lysates (5 μg) were subjected to 10% SDS-PAGE gels and transferred to polyvinylidene difluoride (PVDF) membranes at 80 mV for 1 h. The membranes were incubated overnight at 4 °C with blocking buffer containing 5% skim milk in Tween-Tris buffered saline (TTBS), then were washed for 5 min three times with TTBS and incubated with rabbit polyclonal antibodies (IgG) to *Escherichia coli* GPDH conjugated with horseradish peroxidase (HRP) (LifeSpan BioSciences, Inc., Seattle, WA, USA) in TTBS buffer overnight. Finally, the membranes were washed with TTBS and detected by an enhanced chemiluminescence (ECL) kit (Pierce, Rock-ford, IL, USA). To detect antibodies to α-hemolysin in *S. aureus*-immunized mice, recombinant α-hemolysin (10 μg) was subjected to a 10% SDS-PAGE for Western blot analysis using sera from *S. aureus*-immunized mice and HPR-conjugated rabbit polyclonal IgG.

1.2.7. Antibody Detection and Test Strip Fabrication

For quantification of antibody titers, the lysates of *S. aureus* or recombinant proteins (0.1 μg/well) diluted in 100 μL PBS were coated onto a 96-well microplate at room temperature overnight. After blocking with 2% skim milk in PBS at room temperature for 1 h, diluted sera from mice were added to the wells and incubated for 2 h. A goat anti-mouse IgG (H+L) IgG–HRP conjugate (Promega, WI, USA) (1:10,000 dilution) was added and incubated for 1 h. HRP activity was determined with an OptEIA™ Reagent Set (BD). The OD of each well was measured at 450 nm subtracted from 570 nm ($OD_{570-450}$). For fabrication of test strips, an immunochromatographic strip, which consists of three pads (sample, conjugate release, and absorbent pads), and one nitrocellulose [7] membrane with test and control zones was created. The gold nanoparticle pad (GP) and sample pad (SP) were used to assembly an immunochromatographic strip. Test (T) and control (C) zones on NC membranes were created by spotting BSA, recombinant α-hemolysin, lysates of *S. aureus* or USA300 (lower position), and rabbit anti-mouse IgG secondary antibody (upper position). The sample pad of an immunochromatographic strip was soaked into 2.5% sera (200 μL) of *S. aureus*-immunized mice.

1.2.8. Passive Immunization of *S. aureus* or α-Hemolysin against USA300 Infection

Complements in the sera were inactivated by heating at 58 °C for 30 min. USA300 (10^8 CFU/10 μL) was pre-treated with 5 % (*v*/*v*) inactivated anti-serum to GFP, *S. aureus* or α-hemolysin in the media at 37 °C for 1 h. The USA300 bacteria with serum were then applied onto a 1 cm wound on the dorsal skin of ICR mice. Three days after application, the lesion sizes, the number (CFUs) of USA300 and the level of pro-inflammatory MIP-2 cytokine were quantified as described above.

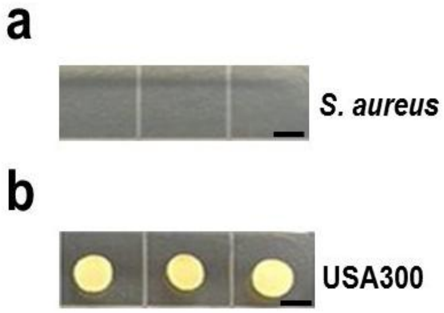

Figure S1. USA300 selected agar plates supplemented with benzylpenicillin. Commensal *S. aureus* (**a**) or USA300 (**b**) (10^7 CFU/mL) was spotted on benzylpenicillin (32 μg/mL)-containing TSB plates in triplicate overnight. Bars = 0.5 cm.

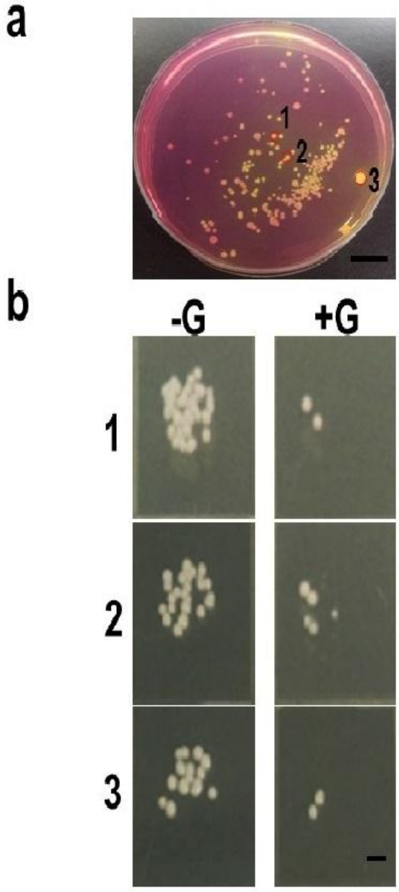

Figure S2. Interference of USA300 growth by three colonies of commensal *S. aureus*. Skin bacteria were isolated from skin around the nose of a healthy male subject without infection and immediately inoculated onto a MSA plate overnight. (**a**) Three colonies labeled 1, 2, and 3 with yellow zones (circles) were identified as commensal *S. aureus*; (**b**) Commensal *S. aureus* (colony 1, 2, or 3) (10^7 CFU/mL) was co-cultured with USA300 (10^5 CFU/mL) in rich media (10 mL) in the presence (+G) or absence (−G) of glycerol (20 g/L). After a 4-day culture, media (5 μL) were spotted on benzylpenicillin (32 μg/mL)-containing TSB plates overnight. Bars = 0.5 cm.

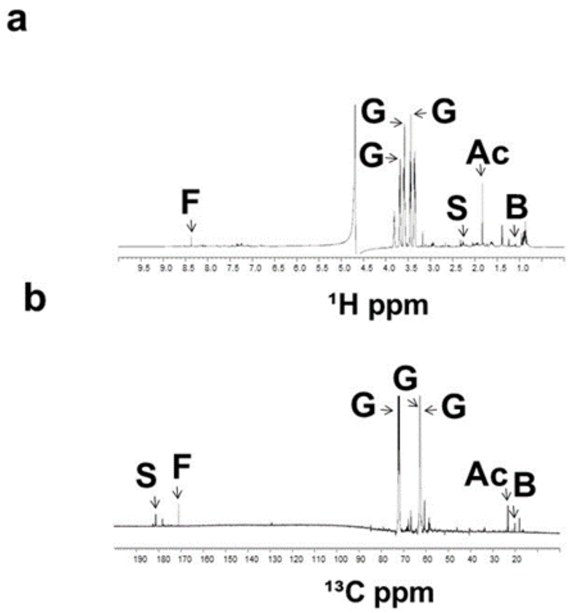

Figure S3. SCFA identification by NMR analysis. SCFAs in the supernatants of fermented media of commensal *S. aureus* are displayed in the 1-D 1H- (**a**) and 13C- (**b**) NMR spectra (400 MHz) after incubation with 13C3-glycerol for four days. G, glycerol; Ac, acetic acid; B, butyric acid; S, succinic acid.

ATGTTGAACGTCGTTCCCTCTAGAATAATTTTGTTTAACTTTAAGAAGGAGATATACATATG
GCTAGCATGACTGGTGGACAGCAAATGGGTCGGGATCCGAATTCCATGAAAACACGTATA
GTCAGCTCAGTAACAACAACACTATTGCTAGGTTCCATATTAATGAATCCTGTCGCTAATGC
CGCAGATTCTGATATTAATATTAAAACCGGTACTACAGATATTGGAAGCAATACTACAGTA
AAAACAGGTGATTTAGTCACTTATGATAAAGAAAATGGCATGCACAAAAAAGTATTTTATA
GTTTTATCGATGATAAAAATCATAATAAAAAACTGCTAGTTATTAGAACGAAAGGTACCAT
TGCTGGTCAATATAGAGTTTATAGCGAAGAAGGTGCTAACAAAAGTGGTTTAGCCTGGCCT
TCAGCCTTTAAGGTACAGTTGCAACTACCTGATAATGAAGTAGCTCAAATATCTGATTACTA
TCCAAGAAATTCGATTGATACAAAAGAGTATATGAGTACTTTAACTTATGGATTCAACGGT
AATGTTACTGGTGATGATACAGGAAAAATTGGCGGCCTTATTGGTGCAAATGTTTCGATTGG
TCATACACTGAAATATGTTCAACCTGATTTCAAAACAATTTTAGAGAGCCCAACTGATAAA
AAAGTAGGCTGGAAAGTGATATTTAACAATATGGTGAATCAAAATTGGGGACCATATGAT
AGAGATTCTTGGAACCCGGTATATGGCAATCAACTTTTCATGAAAACTAGAAATGGCTCTA
TGAAAGCAGCAGATAACTTCCTTGATCCTAACAAAGCAAGTTCTCTATTATCTTCAGGGTTT
TCACCAGACTTCGCTACAGTTATTACTATGGATAGAAAAGCATCCAAACAACAAACAAAT
ATAGATGTAATATACGAACGAGTTCGTGATGACTACCAATTGCACTGGACTTCAACAAATT
GGAAAGGTACCAATACTAAAGATAAATGGATAGATCGTTCTTCAGAAAGATATAAAATCG
ATTGGGAAAAGAGAAATGACAAATCTCGAGCACCACACACACACTGAGATCGCTGCTAAC
AAAGCCCGAAAGAGCTGAGTGCTGCTGCACGGCTGAGCATACTAGCATACCCTTGGGGCTC
TAAACGGTCTGAGGGTTTTGCTGAGAGAACTAATTCCGATGCGATGGACGCGCCCTGTTAA
GCGCGGCGCGCCAATTAAAG

Figure S4. The nucleotide sequences of α-hemolysin. Molecular cloning of α-hemolysin of commensal *S. aureus* was described in Materials and Methods. The nucleotide sequences of a gene (accession number: Q6SV31) encoding α-hemolysin were illustrated.

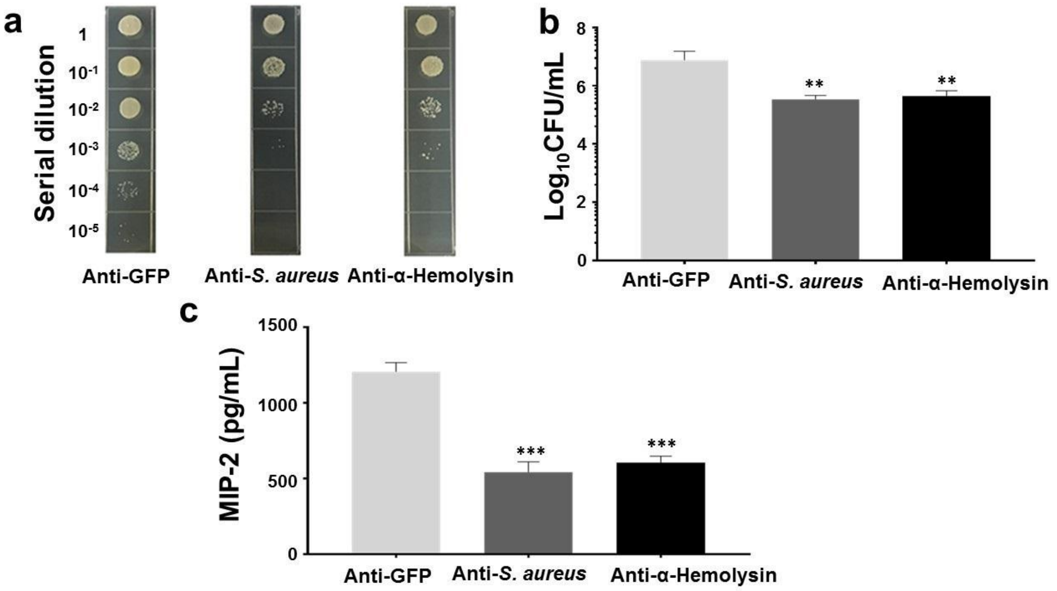

Figure S5. Suppression of USA300 growth in skin wounds by anti-serum to commensal *S. aureus* or *α*-hemolysin. USA300 (10^8 CFU) pre-treated with 5% inactivated anti-serum to GFP, *S. aureus* or *α*-hemolysin were applied onto a skin wound of ICR mice for three days. Bacterial CFUs in the skin wounds were counted by plating serial dilutions (1:10^1 to 1:10^5) of the homogenate on a plate (a). The number ($\log_{10}$ CFU/mL) of USA300 (**b**) and the level of pro-inflammatory MIP-2 cytokine (**c**) were measured. ** $p < 0.01$; *** $p < 0.001$ (two-tailed *t*-tests). Data represent the mean ± SD from results obtained from three independent experiments.

Table S1. Six enzymes (red) involved in the pathway of glycerol fermentation have higher abundance in commensal *S. aureus* than in USA300, while the levels of four enzymes (blue) in commensal *S. aureus* are lower than those in USA300.

Accession#	Protein Names	Ratio of *S. aureus*/USA300
A6U1U2	Glycerol-3-phosphate dehydrogenase (NAD(P)+) (GPDH)	3.77
A6U497	Quinone oxidoreductase putative YhdH/YhfP (NQO)	2.32
A6U492	Malate--quinone oxidoreductase (MQO)	2.00
A6U0W6	Succinate dehydrogenase and fumarate reductase iron-sulfur protein (FRD)	1.17
A6TZQ3	Triosephosphate isomerase (TPI)	0.33
A6U2F4	Glyceraldehyde-3phosphate dehydrogenase, type I (GAPDH)	1.24
A6TZQ2	Phosphoglycerate kinase (PGK)	0.95
A6TYM8	Phosphoglycerate mutase (PGM)	11.42
A6U2G4	Pyruvate kinase (PYK)	0.78
A6TZ66	Phosphate acetyltransferase (PTA)	0.44

References

1. Lindh, J.M.; Terenius, O.; Faye, I. 16S rRNA gene-based identification of midgut bacteria from field-caught Anopheles gambiae sensu lato and *A. funestus* mosquitoes reveals new species related to known insect symbionts. *Appl. Environ. Microbiol.* **2005**, *71*, 7217–7223.

2. Shu, M.; Wang, Y.; Yu, J.; Kuo, S.; Coda, A.; Jiang, Y.; Gallo, R.L.; Huang, C.-M. Fermentation of Propionibacterium acnes, a commensal bacterium in the human skin microbiome, as skin probiotics against methicillin-resistant Staphylococcus aureus. *PLoS ONE* **2013**, *8*, e55380.
3. Wang, Y.; Kuo, S.; Shu, M.; Yu, J.; Huang, S.; Dai, A.; Gallo, R.L.; Huang, C.-M. Staphylococcus epidermidis in the human skin microbiome mediates fermentation to inhibit the growth of Propionibacterium acnes: Implications of probiotics in acne vulgaris. *Appl. Microbiol. Biotechnol.* **2014**, *98*, 411–424.
4. Wiśniewski, J.R.; Rakus, D. Quantitative analysis of the Escherichia coli proteome. *Data Brief* **2014**, *1*, 7–11.
5. Lavallée-Adam, M.; Rauniyar, N.; McClatchy, D.B.; Yates III, J.R. PSEA-Quant: A protein set enrichment analysis on label-free and label-based protein quantification data. *J. Proteome Res.* **2014**, *13*, 5496–5509.
6. Park, S.K.; Venable, J.D.; Xu, T.; Yates, J.R. A quantitative analysis software tool for mass spectrometry–based proteomics. *Nature Methods* **2008**, *5*, 319–322.
7. Rayner, C.; Munckhof, W. Antibiotics currently used in the treatment of infections caused by Staphylococcus aureus. *Intern. Med. J.* **2005**, *35*, S3–S16.
